# With Exposure Comes Familiarity: Older Adults’ Contributions to the Age-Friendly Classroom

**DOI:** 10.1093/geroni/igaa057.1788

**Published:** 2020-12-16

**Authors:** Ashley Ermer

**Affiliations:** Montclair State University, Montclair, New Jersey, United States

## Abstract

Prior research has documented the benefits of intergenerational contact for both younger and older adults (e.g., Christian, Turner, Holt, Larkin, & Cotler, 2014). This presentation will focus on how older adults have contributed to students’ classroom experiences in an upper-level, undergraduate aging policy course. In this course, older adults have previously served as guest speakers, sharing their knowledge about the creation of nonprofit organizations, and their experiences aging within the family. Older adults have also participated in an activity addressing ageism with students from the aging policy course. Feedback from students has largely been positive, with over half of students during stating that they had a better understanding of older adults, that they learned from the experience, and that viewed the intergenerational contact as helpful. Ways in which this model can be extended to other academic disciplines will also be explored.

